# Cadmium induces lung inflammation independent of lung cell proliferation: a molecular approach

**DOI:** 10.1186/1476-9255-6-19

**Published:** 2009-06-12

**Authors:** Subhadip Kundu, Suman Sengupta, Soumya Chatterjee, Soham Mitra, Arindam Bhattacharyya

**Affiliations:** 1 Department of Zoology, University of Calcutta, 35, Ballygange Circular Road, Kolkta-700019, India; 2 Department of Environmental Science, University of Kalyani, West Bengal-741235, India

## Abstract

**Background:**

Cadmium is one of the inflammation-related xenobiotics and has been regarded as a potent carcinogen. The relationship between inflammation and cell proliferation due to chronic infection has been studied, but the mechanism is not fully clear. Though the mode of cadmium toxicity is well characterized in animal cells, still it requires some further investigations. Previously we reported that cadmium induces immune cell death in Swiss albino mice. In the present study we showed that instead of inducing cell death mechanism, cadmium in low concentration triggers proliferation in mice lung cell and our results reveals that prior to the induction of proliferation it causes severe inflammation.

**Methods:**

Swiss albino mice were treated with different concentrations of cadmium to determine the LD50. Mice were subdivided (5 mice each) according to the exposure period (15, 30, 45, 60 days) and were given sub lethal dose (5 mg/Kg body weight) of cadmium chloride and ibuprofen (50 mg/Kg body weight, recommended dose) once in a week. SEM and histology were performed as evidence of changes in cellular morphology. Inflammation was measured by the expression of Cox-2 and MMPs. Expression of proinflammatory cytokines (Cox-2, IL-6), signaling and cell cycle regulatory molecules (STAT3, Akt, CyclinD1) were measured by western blot, ELISA and immunoprecipitation. Mutagenecity was evidenced by comet assay. Cell proliferation was determined by cell count, cell cycle and DNA analysis.

**Results:**

Prolonged exposure of low concentration of cadmium resulted in up regulation of proinflammatory cytokines and cell cycle regulatory molecules. Though NSAIDs like Ibuprofen reduces the expression of inflammatory cytokines, but it did not show any inhibitory effect on cadmium adopted lung cell proliferation.

**Conclusion:**

Our results prove that cadmium causes both inflammation and cell proliferation when applied in a low dose but proliferative changes occur independent of inflammation.

## Background

Cadmium has been shown to have various detrimental effects on health [1]. Upon absorption, Cadmium is rapidly transported by blood to different organs in the body where its estimated half-life in humans is 15–20 years [2]. Additionally chronic exposure to Cadmium has been associated with a number of physiological consequences such as renal failure and immunosuppression as well as various types of cancers in mammals. Several toxicities such as hepatoxicity, neurotoxicity and cardiotoxicity are also documented under high Cadmium exposure [3,4]. In recent years progress has been made in dissecting apart the molecular mechanisms underlying the effects of exposure to this toxic metal.

The amount of Cadmium absorbed in the body following its exposure varies depending on the route of entry. Though the primary routes of cadmium exposure in humans are via inhalation from such sources as cigarette smoking [5], food is also reported as source for human exposure to Cadmium. Cadmium is selectively taken up by certain edible plants and certain food items, such as crab contains Cadmium as high as 30–50 ppm [6]. In general, exposure of cells to low, micromolar concentrations of Cadmium results significant toxicity [7,8]. Strong evidence, based on experimental studies exists to support the carcinogenic potential of Cadmium. Following various routes of exposure to Cadmium, experimental animals produce tumors of multiple organs [9,10]. Only about 5% of a given dosage of cadmium is absorbed from the gastrointestinal tract, while lung absorption is as much as 90% of a dose inhaled into the lungs. Despite being one of the major routes for cadmium absorption, the toxic mechanism of cadmium on lung tissue is still poorly understood [11].

Cadmium induced lung injuries have been recently identified which indicates that it provokes lung damage and inflammation [12] by involving cytokine production [13]. Cadmium-adapted alveolar epithelial cells are protected from oxidant-induced apoptosis along [14] with the expression of the numerous genes in acute-phase proteins or inflammatory cytokines [15]. The acquired self tolerance to Cadmium is thought to have some basis in toxicokinetics but primarily concerns with modified tissue responses [16]. Cadmium is one of the inflammation-related xenobiotics and its exposure on the tissues is often accompanied with infiltration of inflammatory cells [17]. Interleukin, such as IL-6 has a key role in the proliferation of lung cell [18] and Cox-2 is an inducible inflammatory enzyme plays an important role in the progression of human lung adenocarcinoma [19]. Although Cox-2 expression in tumors increases angiogenesis, which is highly associated with induction of various growth factors like IL-6 [20]. Various studies reported that cadmium promotes lung cell proliferation by an immune suppressive network which involves over expression of Cox-2 [21]. On the other hand IL-6 and its receptor interactions activate STAT3 which in turn induce the expression of several anti apoptotic proteins and thereby promotes cell proliferation. It is reported that cell expresses elevated levels of CyclinD1 when stably transfected with a dominant-active STAT3 construct [22]. Therefore the general mode of action of all these signaling molecules are either directly related to the inflammation only or to the development of cell proliferation influenced by chronic inflammation. Although cadmium exposure has been reported to cause neoplastic transformation of human prostatic epithelial cells, but the efficacy of this transformation is highly dependent on the dose of the metal ion [23,24]. Exposure of normal human prostate epithelial cells to higher dose (10 μM) cadmium transiently increased the expression of *p53*, *c-myc*, and *c-jun *after 2 hr as a prelude to apoptosis [25] where as lower dose promotes proliferation and resistance to apoptosis.

We are particularly interested in the contribution of low doses (5 mg/kg body weight) of cadmium to the transformation of mice lung. We have found that, due to cadmium exposure, expression of IL-6, STAT3 and inflammatory enzyme MMP-2, Cox-2 increased significantly. Also we have the evidences that increased activity of the Akt signaling axis in lung cells appears to operate in conjunction with or parallel to increased STAT3 activation to induce proliferation programme. We showed that cadmium promotes lung inflammation and cell proliferation both in independent manner. Thus, this study was designed to determine the effects of chronic exposures of low concentration of Cadmium in vivo. It is not worthy that anti-inflammatory drug treatment could not totally inhibit the proliferation process, whereas inflammation was prevented.

## Methods

### Chemicals and reagents

Antibodies against Cyclooxigenase-2 (Cox-2), IL-6, STAT-3, p-STAT3, Akt, p-Akt, CyclinD1, β-actin and anti-mouse-AP, anti-rabbit AP antibodies was obtained from Cell Signaling Technology, Inc. USA. Prestain molecular weight protein marker and standard DNA ladder, goat anti mouse-HRP and anti rabbit-HRP conjugated antibody and DAB developing system for immunohistochemistry were purchased from Bangalore Genei (India). Gelatin substrate for MMP was purchased from Sigma USA. Ibuprofen and the remaining chemicals were purchased from local firms (India) and were of highest purity grade.

### Animal models and treatment

All animal experiments were performed following "Principles of laboratory animal care" (NIH publication No. 85-23, revised in 1985) as well as specific Indian laws on "Protection of Animals" under the provision of authorized investigators. Swiss albino mice (~25 g each; 5 mice in each group) were randomly divided into three groups such as (i) Control set, (ii) Cadmium (5 mg/Kg body weight) treated set, (iii) Cadmium and Ibuprofen (50 mg/Kg body weight) treated set. These sets were again divided into four groups according to the time dependent exposure (like 15 days, 30 days, 45 days, 60 days) of Cadmium and Ibuprofen. Cadmium chloride and Ibuprofen were dissolved in sterile pyrogen-free saline water and were given as i.p injection. Control set were given sterile pyrogen-free saline water only as i.p [26].

### Histological analysis

Lungs were taken after treatment and were washed in PBS immediately. The tissues were fixed for 24 hours in buffered formaldehyde solution (10% in PBS) at room temperature, dehydrated by graded ethanol and embedded in Paraffin (MERCK, Solidification point 60–62°C, TEST CAS No-8002-74-2). Tissue sections (thickness 5 μm) were deparaffinized with xylene, stained with eosin/haematoxylin, Digital images were captured with Olympus CAMEDIA digital camera, Model C-7070 wide zoom (100×) [27].

### Preparation of Cytosol

Lung were homogenized in homogenizing buffer (0.25 M sucrose, 5 mM HEPES buffer, and 1 mM EDTA, pH 7.2), containing protease inhibitor PMSF (SRL, India). The homogenate was centrifuged at 500 × g to pellet nuclei and the resulting supernatant was centrifuged at 100,000 × g (29,000 rpm) for 60 min at 4°C in a 50.2 Ti rotar. Cytosolic supernatant was collected and aliquots were frozen by immersion in liquid nitrogen. It was stored at -80°C until use.

### Zymographic Analysis of MMP Activity

Cytosolic extracts of lungs were prepared and protein concentration was determined by Bradford method. Gelatinase zymography was performed in 10% NOVEX Pre-Cast SDS Polyacrylamide Gel (Invitrogen Corp.) in the presence of 0.1% gelatin under non reducing conditions. Protein lysate of lungs were mixed with sample buffer and loaded for SDS-PAGE with tris glycine SDS buffer as suggested by the manufacturer (Novex). Samples were not boiled before electrophoresis. Following electrophoresis the gels were washed twice in 2.5% Triton X-100 for 30 min at room temperature to remove SDS. The gels were then incubated at 37°C overnight in substrate buffer containing 50 mM Tris-HCl and 10 mM CaCl2 at pH 8.0 and stained with 0.5% Coomassie Blue R250 in 50% methanol and 10% glacial acetic acid for 30 min and destained. Upon renaturation of the enzyme, the gelatinases digest the gelatin in the gel and give clear bands against an intensely stained background. Protein standards were run concurrently and approximate molecular weights were determined by plotting the relative mobilities of known proteins. After destaining, the bands were quantified using the UVTtech software (GAS 9500/9511, SRL No-0710161, Cambridge) [28].

### ELISA

Lungs were dissected out after the stimulation of 5 mg/Kg body weight Cadmium for two months and were homogenized in homogenizing buffer, and then supernatants were collected and stored at -80°C. Cox-2 and IL-6 in the supernatants were measured by ELISA according to the manufacturers' instructions (Cell Signaling manual).

### Immunohistochemistry

Sections (5 μm) were cut from paraffin embeddedtissues and mounted on positively charged Super frost slides (Export Mengel CF). Tissues were deparaffinized, rehydrated through graded alcohols, and then blocked for endogenous peroxidase in 3% hydrogen peroxide in methanol. All tissues were preblocked in Tris-buffered saline containing 0.3% Triton, and 0.5% blocking agent (BSA, SRL, India, and batch No-832095) and incubated with Cox-2 (Cell signaling Technologies, USA), primary antibody (1:30) overnight at 4°C for positive control. Antisera specific for Cox-2 were diluted 1:30 in Tris-buffered saline containing 0.3% Triton, and 0.5% blocking agent. Immunoreactive complexes were detected using DAB system (Bangalore GeNei DAB system, Cat #SFE5). Slides were counterstained briefly in haematoxylin (MERCK), mounted in DPX (MERCK). Slides for negative control were treated with no primary antibody.

### Western blot analysis

For western blot analysis of IL-6, p-Akt, Cox-2, p-STAT3 and CyclinD1, cell lysate was loaded into a 10%–15% SDS-polyacrylamide gel. After electrophoresis the gel was transferred to nitrocellulose membrane and blocked with nonfat dry milk in TBS containing Tween-20. Each primary antibody was diluted at 1:1000 ratio in TBS and after overnight incubation, the membrane was again blocked with nonfat dry milk in TBS containing Tween-20. Secondary antibody was diluted at 1:1000 ratio and after 2 hours incubation the membrane was developed by NBT/BCIP (HIMEDIA, Cat# RM 578, RM 2577). β-actin was chosen for constitutive expression.

### Immunoprecipitation

For immunoprecipitation, cleared lysate was prepared and about 100 μg of protein were immunoprecipitated using 10 μl of anti-cdk4 (Cell signaling Technologies, USA) for overnight at 4°C with gentle rotation. 25 μl Protein G CL-Agarose (Bangalore Genei, India, Cat # LIA 43S) was added to the previous mixture, depending on the experiment and allowed it to mix for 4 hours at 4°C with gentle rotation. It was then centrifuged at 3000 rpm for 2 min. The immunoprecipitates were washed extensively with sterile PBS and separated by SDS-PAGE, followed by western analyses with anti CyclinD1 antibody (Cell signaling Technologies, USA) as described above.

### Isolation of the lung cell & cell viability assay

Lung was removed from mice aseptically followed by addition of collaginase. Single cell suspensions were made in RPMI 1640 by passing the cell population through a nylon mesh with 50 μm pore size. The leukocyte population was then allowed to adhere in Petri dishes at 37° for 1 hour. The non-adherent cell populations were collected and subjected to Ficoll-Hypaque density gradient separation. The buffy layer was collected, washed and used as the source of cells. Viable lung cells were counted in haemocytometer by trypan blue exclusion test and used for further analysis.

### Comet assay

Comet assays were performed under alkaline conditions to determine the amount of double-strand DNA breaks. Lungs from the mice treated with different concentrations of Cadmium chloride were trypsinized and washed in PBS before being added to preheated (37°C) low-melting point agarose. The solution was pipetted onto slides precoated with 1% agarose. The chilled slides were allowed to lyse for 40 min at 4°C in 2.5 M NaCl, 100 mM NaEDTA (pH 10), 10 mM Tris Base, 1% SDS, 1% Triton X-100 prior to immersion in alkaline electrophoresis solution (300 mM NaOH, 1 mM EDTA, pH 13). After 30 min, slides were placed into a horizontal electrophoresis chamber samples for 30 min (1 V/cm at 4°C). The slides were washed with deionized H_2_O to remove the alkaline buffer, dehydrated in 70% ice-cold EtOH and air-dried overnight. Slides were stained with EtBr (50 μg/ml) and examined by microscopy (Lica, China). Tail length (TL) was used to quantify the DNA damage. Image analysis and quantification has been performed with Motic Image software.

### Scanning Electron microscopy

Lungs from normal, Cadmium treated (5 mg/Kg body weight) and Cadmium plus Ibuprofen treated (50 mg/Kg body weight) were dissected out from the mice and were immediately washed into the phosphate buffer saline (PBS) to remove any mucus, blood or any other contaminant. Tissues were fixed with 2.5% glutaraldehyde in 0.1 M phosphate buffer pH 7.2–7.4 for 24 hours. Tissues were then dehydrated with graded Acetone (MERCK) (50%–100%, 20 minutes each). We did not perform the Critical point drying (CPD) step as the lung tissues were very fragile. Sample were cut into small part and placed on the stab and put into the IB-2 ion coater for gold coating. After coating the sample were observed by S530-Hitachi SEM instrument (Department of USIC, University of Burdwan).

### Cell cycle analysis by flowcytometry

Lung cells were fixed with p-formaldehyde, permeabilized with Triton X-100, and nuclear DNA was labeled with propidium iodide (PI). Cell cycle phase distribution of nuclear DNA was determined on FACS (Fluorescence-Activated Cell Sorter) having fluorescence detector equipped with 488 nm argon laser light source and 623 nm band pass filter (linear scale) using Cell Questpro software (Becton Dickinson). Total 10,000 events were acquired and flowcytrometric data were analyzed using Cell Questpro software.

### DNA analysis

For DNA laddering assay, lungs were first placed into liquid nitrogen for immediate hardening. It was crushed then into powder and followed the extraction protocol as described in Molecular Cloning, Vol-1, Sambrook, Russell (Chapter-6, Unit-6.4).

### Statistical Analysis

Values are shown as Standard error of mean, except where otherwise indicated. Data were analyzed and, when appropriate, significance of the differences between mean values was determined by the Student-T test. Results were considered significant at p < 0.05.

## Results

### Determination of LD50 and dose for experiments

**Figure 1:**
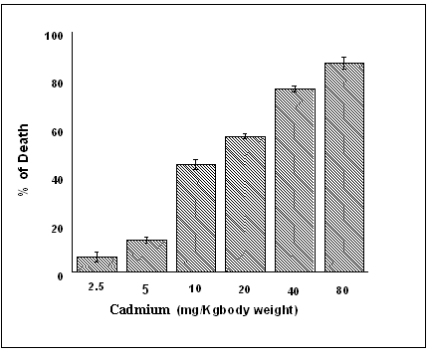
**Dose dependence survival of Cadmium treatment**. Each value represents the percentage of died populations in each dose chosen with +/- SEM (n = 5) and p < 0.05, compared to normal. The experimental groups represent the Cadmium treated mice [inject CdCl_2_, (i.p.)] at a concentration of 2.5 mg/Kg body weight, 5 mg/Kg body weight, 10 mg/Kg body weight, 20 mg/Kg body weight and 40 mg/Kg body weight and 80 mg/Kg body weight respectively. The sub lethal concentration (5 mg/Kg body weight) was chosen as an applied dose.

To determine the LD50, mice were administered different concentration of Cadmium (2.5, 5, 10, 20, 40 and 80 mg/Kg body weight) till 60 days period (single dose in a week). It was found that 50% of the experimental population died at a concentration of about 10 mg/Kg body weights, (Fig. 1) and there is a significant decrease (p < 0.05) of survival according to the increasement of dose. Therefore 5 mg/Kg body weight (sub lethal dose) concentration was chosen for further experiments to elaborate the intricate mechanisms.

### Prolonged exposure of low dose of Cadmium induces lung oedema and inflammation

**Figure 2:**
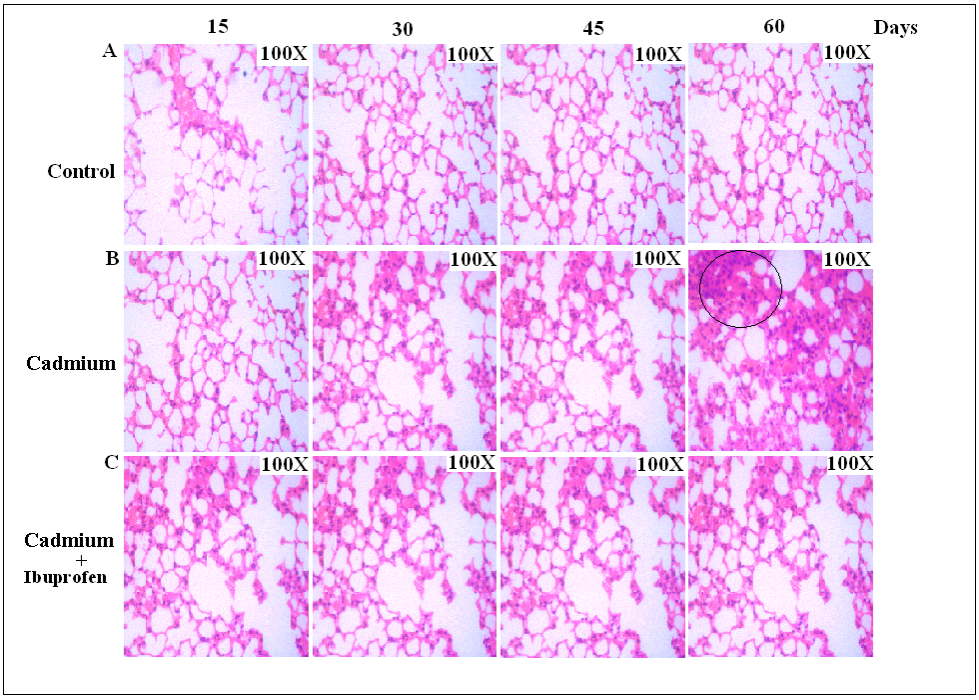
**Histopathology of lung sections of Swiss albino exposed to Cadmium (5 mg/Kg body weight) showing progressive lung inflammation and prevention by Ibuprofen**. A, Histology of lung sections of normal mice. B, Mice were treated with low dose of Cadmium (5 mg/Kg body weight), subsequently for 15, 30, 45, 60 days, granulation and air space enlargement was shown after 45 days which is the sign of inflammatory development. C, Application of Ibuprofen reduces the chance of lung oedema formation throughout the experimental periods. Original magnification (100×).

To evaluate the effect of low concentration of Cadmium on mice lung cell, we pulsed the mice with 5 mg/kg body weight Cadmium for different time interval subsequently for 15 days, 30 days, 45 days, and 60 days. No lung lesion or oedema was shown to develop upto 45 day. Histological analysis revealed that appearance of alveolar oedema and inflammation after prolonged cadmium exposure along with the airspace enlargement after 8 -week of cadmium exposure (Fig. 2B). At the same time, mice those who were exposed to same dose of cadmium chloride along with non steroidal anti-inflammatory drug like Ibuprofen (50 mg/kg body weight), showed reduced level of inflammatory development (Fig. 2C) while compared to the normal (Fig. 2A). This data suggests that cadmium if applied even in a very low concentration for a long period of time can induce inflammation in experimental animals.

### Zymography proves MMP-2 but not MMP-9 is one of the mediators of such type of inflammation

**Figure 3:**
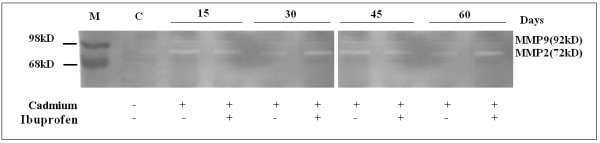
**SDS-PAGE and Gelatin zymography represent the expression of MMP-2**. Detection, by gelatin zymography, of matrix metalloproteinase-2 (MMP-2) and (MMP-9) expression at different time points after the induction of Cadmium (5 mg/Kg body weight). Lung cell extracts were prepared at days of 15, 30, 45 and 60 from normal (N), Cadmium treated and Cadmium plus Ibuprofen treated (three individual animals per dose) mice. The zymography was developed and stained as described in Materials and Methods. The picture shows increased expression of MMP-2(72 kD), which was not inhibited by Ibuprofen. Gel is representative of three comparable experiments indicate p < 0.05 with respect to the control.

There are different mechanisms are thought to be responsible for the development and progression of cadmium induced inflammation in lung. Increased secretion and/or activity of matrix metalloproteinases (MMP), especially MMP-2 and MMP-9, have been identified in inflammatory cells and tissues isolated from human suffering from COPD [29]. In order to find out the mechanism of cadmium induced lung inflammation we performed the zymograph analysis to measure the matrix metalloproteinase expression. We observed significant expression of MMP-2 but not MMP-9 (Fig. 3) throughout the experimental period. Though NSAID like Ibuprofen is sufficient enough in suppressing the expression of matrix metalloproteinases, but it failed to operate its inhibitory action here, p < 0.05 with respect to the control.

### Cyclooxygenase-2, IL-6, p-STAT3 and p-Akt expression are the key regulators of cadmium induced lung inflammation

**Figure 4:**
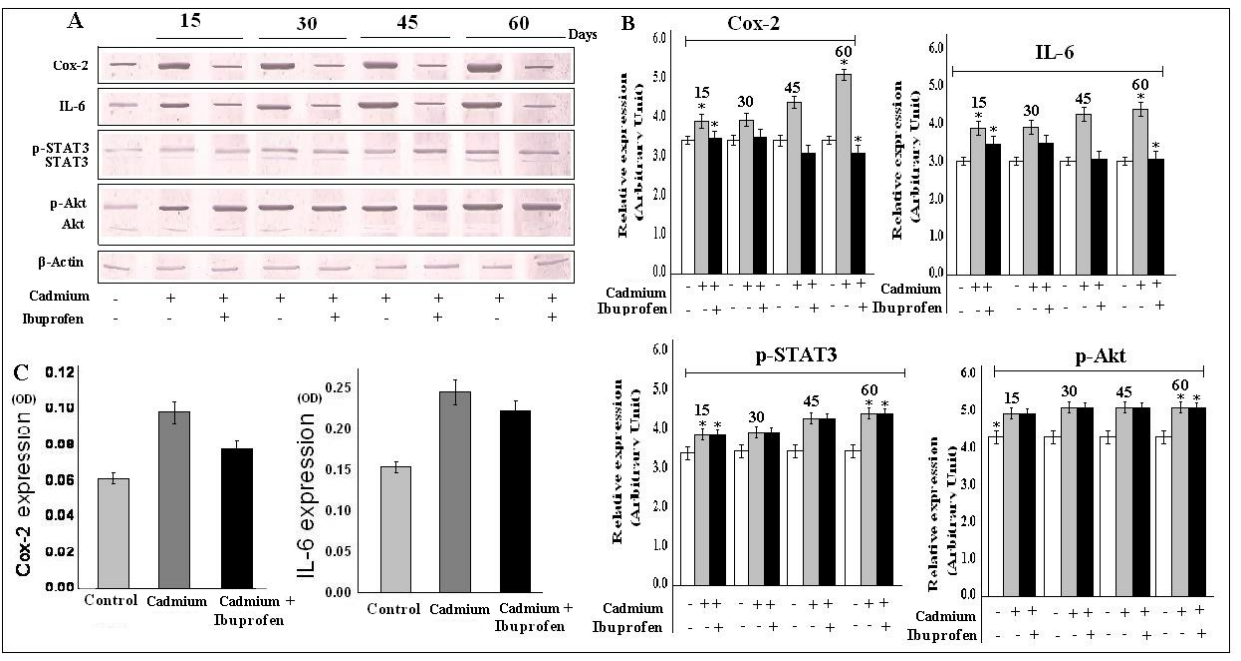
**Cadmium induced expression of Cox-2, IL-6, p-STAT3 and p-Akt**. Cell lysate from control and treatment lung were subjected to western blot analysis and ELISA. A, Expression of Cox-2, IL-6, p-STAT3, p-Akt were increased throughout all the experimental period. Though Ibuprofen reduced the expression of Cox-2 and IL-6, but it could not be able to revert the expression of p-STAT3 and p-Akt. B, Bar graphs represent the quantitative densitometric value of the expressed protein. C, OD values showed significant increasement of Cox-2 and IL-6 expression by ELISA. Data is representative of three comparable experiments and indicate p < 0.05 with respect to the control.

The regulation of Cox-2 is very important in various inflammatory and proliferative responses, which is sometime induced by IL-6 [20] signaling pathway. Therefore, we wanted to see that whether this cadmium induced lung cell proliferation has any relationship with Cox-2 and IL-6 expression and we found that expression of both Cox-2 and IL-6 were increased to a significant level while application of Ibuprofen was able to reduce the expression of Cox-2 and IL-6 (Fig. 4A) and it was strongly supported by our ELISA results (Fig. 4C). STAT3 and Akt are the downstream modulators of the IL-6 and there regulations are important in growth signaling. So we studied the expression of p-STAT3 and p-Akt, following cadmium exposure. In our study, we also observed, that both p-STAT3 and p-Akt expression (but not the normal STAT3 and Akt) were up regulated (Fig. 4A) in treated mice, but application of Ibuprofen could not revert these expression status. The result indicated that cadmium induced cell proliferation through p-STAT3 and p-Akt activation may have inflammation independent pathways. The investigation still under progress (data not shown). Data is representative of three independent experiments (p < 0.05).

### Immunohistochemical expression of Cox-2

**Figure 5:**
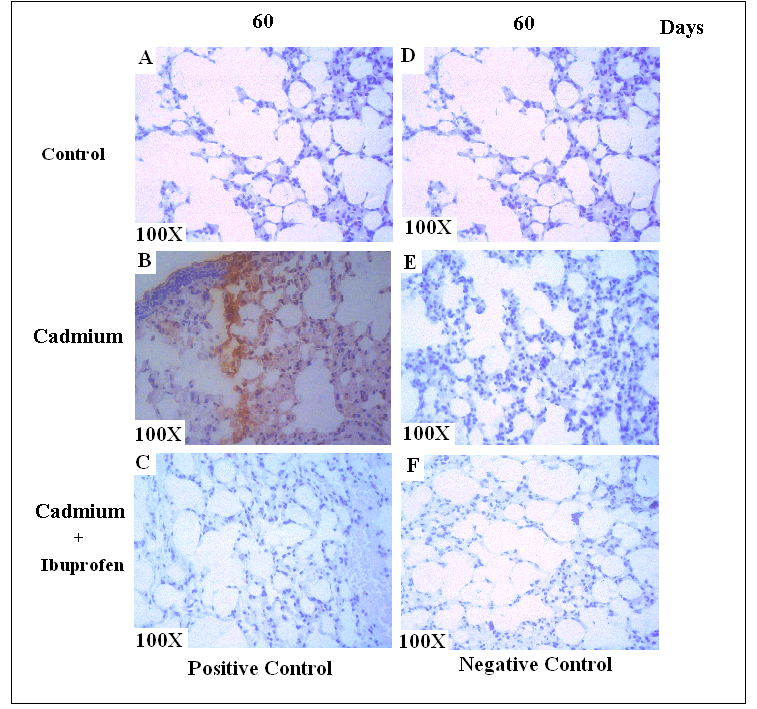
**Immunohistochemical expression of Cox-2**. A, Control lung shows no expression of Cox-2. B, Cox-2 expression was profoundly increased in treated lung. C, Application of Ibuprofen reduces the expression level of Cox-2. D, E, F, Negative staining shows no such expression. Original magnification (100×).

To support our previous result we observed the expression level of Cox-2 by immunohistochemistry. After two months of exposure cadmium treated lung showed increased level of Cox-2 expression (Fig. 5B), and was reduced in Ibuprofen treated lung (Fig. 5C), while compared with the control slide (Fig. 5A). In negative control set, no such expression of Cox-2 was observed (Fig. 5D, 5E, 5F). We observed same result in case of IL-6 expression (data not shown). The result proved that low dose of cadmium causes inflammation of lung cell by inducing the proinflammatory cytokine like Cox-2 and IL-6.

### Low-dose Cadmium exposure increased cell number

**Figure 6:**
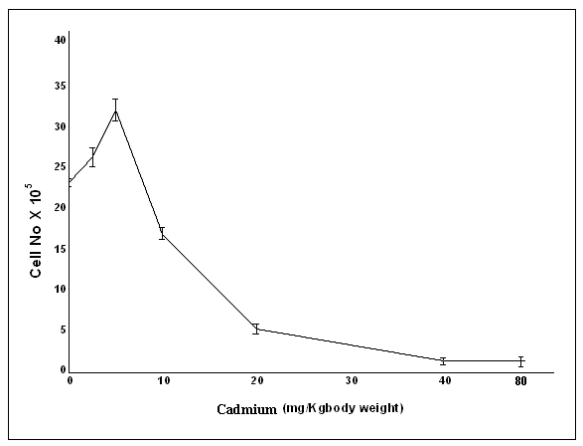
**Cadmium causes lung cell proliferation**. We evaluated the effect of cadmium on the lung cell count in normal and treated mice. The normal and treated mice were given pyrogen free saline water and different dose of Cadmium (2.5 mg, 5 mg, 10 mg, 20 mg, 40 mg and 80 mg/Kg body weight) respectively for two months. Cell count gradually increased in low dose of CdCl_2 _(2.5, 5 mg/Kg body weight), but beyond that (10 mg, 20 mg, 40 mg and 80 mg/Kg body weight) cell death occurred. Data is representative of three independent experiments (p < 0.05).

So far we have proved that Cadmium, when applied in a low concentration (Sub-lethal dose), capable of inducing the expression of some proteins, those are very much involved in stimulating cellular inflammation and proliferation. Therefore, we took the lung cell count after the exposure of different concentrations of Cadmium. We found that cell number increased in mice those were adapted with low dose of Cadmium (5 mg/Kg body weight), but at the same time reduced cell count were followed in mice treated with high dose of Cadmium (10 mg, 20 mg, 40 mg and 80 mg/Kg body weight), when compared with the normal (Fig. 6). This data clearly indicates that chronic cadmium exposure first induced inflammation and then cell proliferation. Data is representative of three independent experiments (p < 0.05).

### DNA damage analysis clearly suggests that cellular effect of cadmium is totally dose dependent

**Figure 7:**
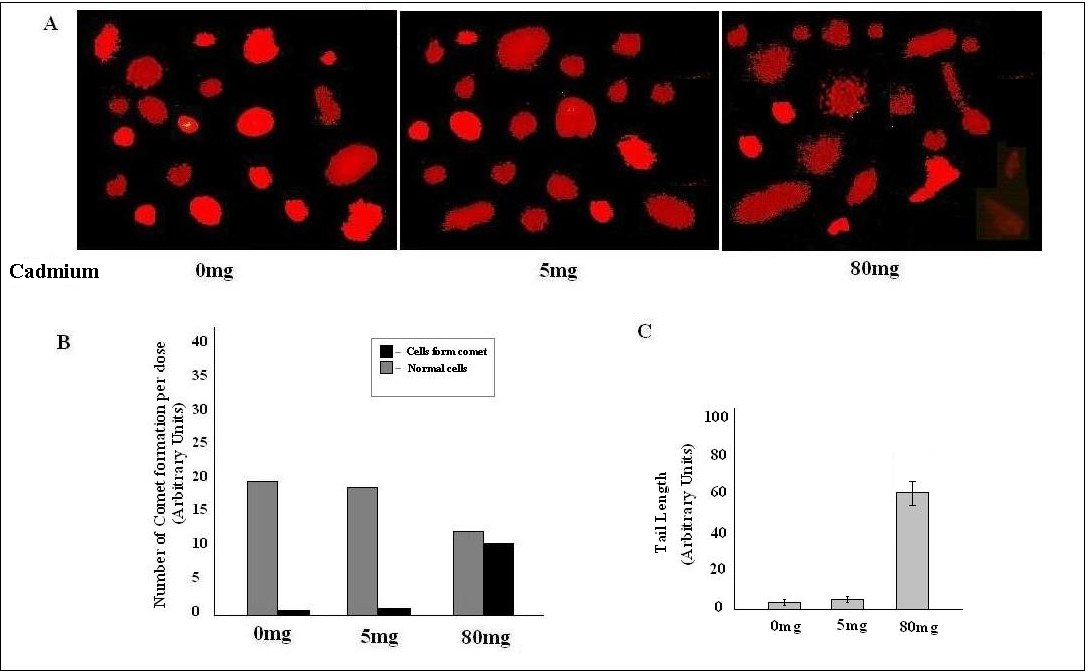
**Comet assay**. A, DNA tail formation was increased in higher dose of Cadmium, while no significant change was observed between control and low dose. B, C, Showed tail formation increased significantly in 80 mg/Kg body weight dose of Cadmium. Data is representative of three independent experiments (p < 0.05).

For further confirmation of our cell count data, we performed single cell gel electrophoresis (Comet assay), and found that low exposure of Cadmium (5 mg/kg body weight) did not show any significant DNA damage. We performed the assay also with high dose (80 mg/kg body weight) of Cadmium, to see whether it shows the same effect or not. We observed that 80 mg/kg body weight Cadmium induces tail formation, which was the indication of DNA damage. The result proved dual role of cadmium chloride, when applied in low dose it causes cell proliferation, otherwise it promotes cell death (Fig. 7A). It is clear from the Fig. 7B and Fig. 7C, that tail formation and cellular deformity were increased along with the application of high CdCl_2 _concentration. Data is representative of three independent experiments (p < 0.05).

### Evaluation of cell cycle pattern after cadmium treatment

**Figure 8:**
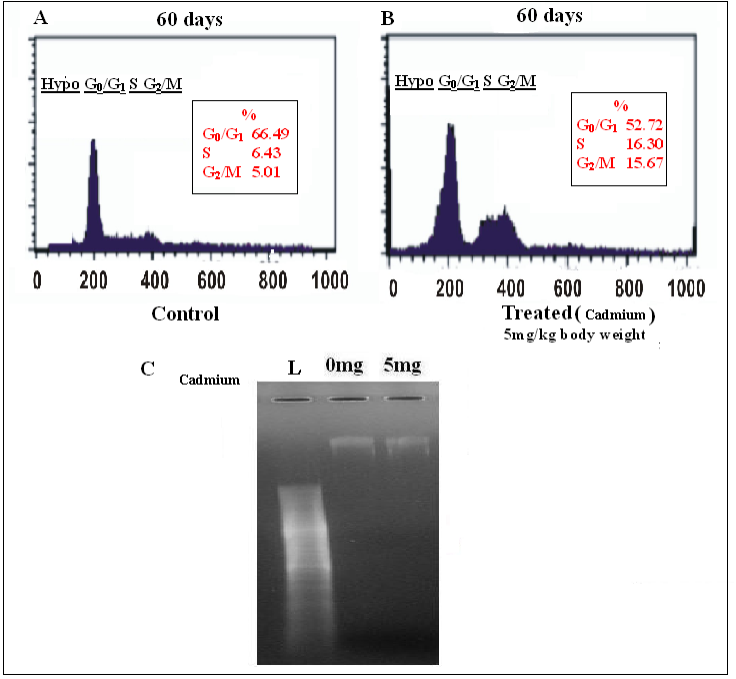
**Effect of Cadmium on cell cycle distribution of mice lung cell determined by flowcytometry analysis**. B, Percentage of cell increased in S-G_2_/M phase in treated set. A, Increased cell number was observed in sub-G_0_/G_1 _phase in control set, which was the indication of cell proliferation. Data is representative of three independent experiments (p < 0.05).C, No DNA ladder was formed in low dose (5 mg/Kg body weight) CdCl_2 _treated lung while compared with control ladder (L) and normal one.

We next studied the cell cycle phase distribution of lung cells treated with Cadmium (5 mg/Kg body weight) for two months (Fig. 8). It is interestingly seen that the results supported our notion and the total DNA content of S-G_2_/M phase in treated (Fig. 8B) group increased by 31.91% compared to that of 11.40% in normal one (Fig. 8A). To further confirm this data, we examined the DNA laddering pattern of the treated lung and compared it with the control set. It is clear from the Fig. 8C that no the ladder was formed in the low cadmium chloride treated lung, which can be correlated with our cell count data and it strongly suggests that Cadmium (low dose) itself by promoting inflammatory responses can produce cellular proliferation in mice lung, which could not be reverted by non steroidal anti-inflammatory drug like Ibuprofen (data not shown). Data is representative of three independent experiments (p < 0.05).

### Scanning Electron Microscopy

**Figure 9:**
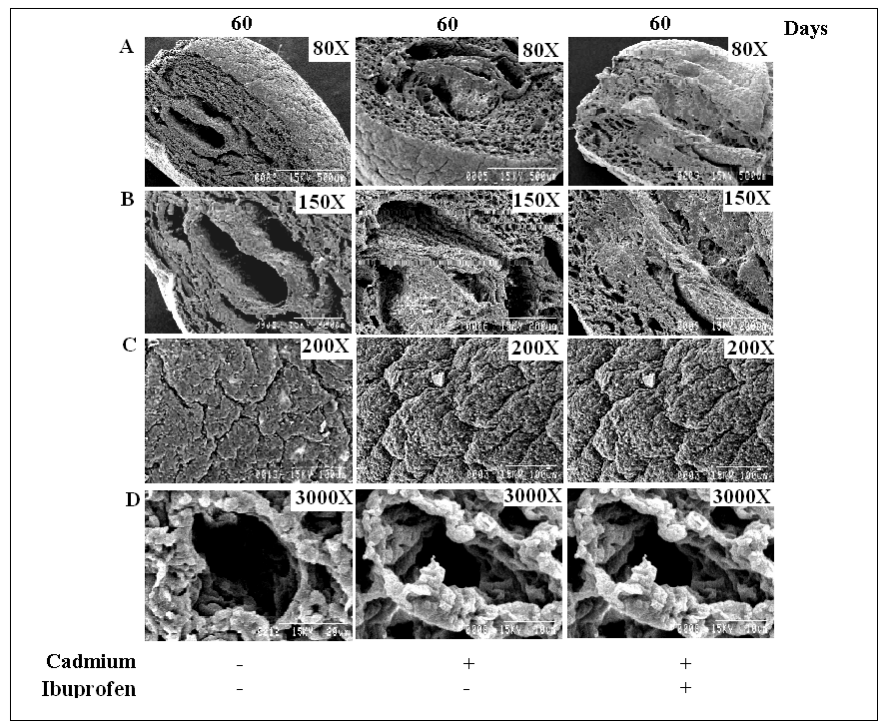
**Scanning Electron Microscopy of Cadmium treated lung**. A, No lobular appearances were observed under lower magnification. B, Alveolar space was getting narrowed in treated lung, compared to the normal one. C, Lobular appearances were prominent at higher magnification. D, Narrow edicular spaces signify the increasement of cell density. Ibuprofen showed no effect to revert the structural deformity.

Our cell count and cell cycle results clearly indicated the instead of inducing cell death mechanism cadmium in low concentration promotes cell proliferation. We observed the surface structure of lung after 60 days by scanning electron microscopy to see whether there is any formation of tumor likes growth or not. We did not observe any tumor pattern on cell surface (Fig. 9A), but architectural distortion of the pulmonary microvasculature was evidenced in treated mice. Alveolar space was getting narrow along with the enlargement epithelial basement (Fig. 9B), which is the indication of acute inflammation. In higher magnification, cell surface became uneven and lobular (Fig. 9C), Narrow edicular spaces signify the increasement of cell density (Fig. 9D). Ibuprofen showed no effect to revert the structural deformity of lung caused by cadmium.

### Cadmium Induced CyclinD1 Regulation in lung Cell

**Figure 10:**
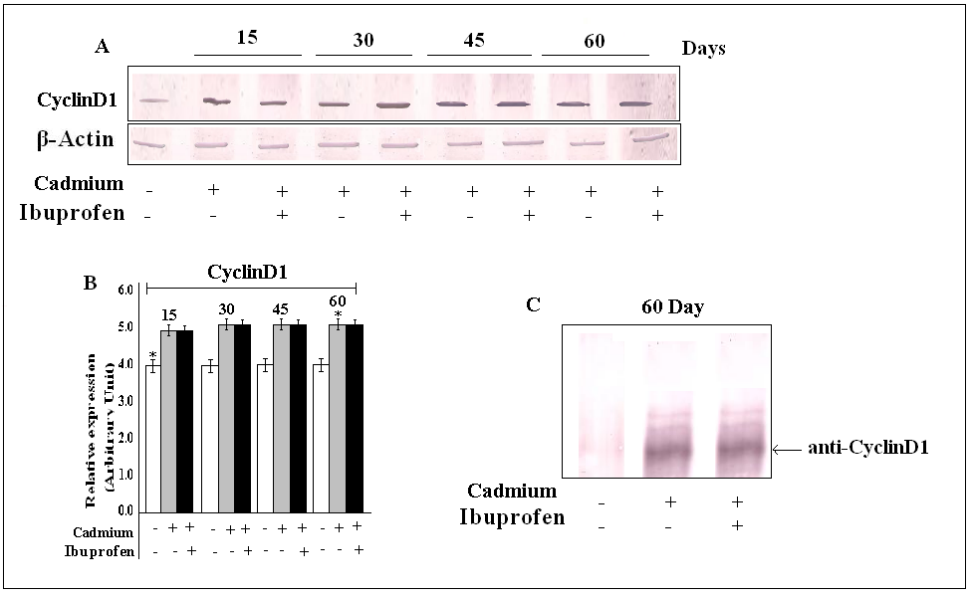
**Effect of Cadmium on CyclinD1 expression**. A, Expression of CyclinD1 was increased throughout all the experimental period. B, Bar graphs represent the quantitative densitometric value of the expressed protein. Data is representative of three comparable experiments indicate p < 0.05 with respect to the control. C, Immunoprecipitation showed the formation CyclinD1-Cdk4 complex, which is an indication of cell cycle progression.

Our previous experiment has proved that Cadmium (5 mg/Kg body weight) challenged lung cell acquired the capacity of proliferation after a chronic exposure. Our cell cycle pattern has revealed that cell number is increased significantly in S and G_2_/M phase along with the reduction in G_0_/G_1 _phase, which is a clear indication of cell proliferation. Therefore, we had to determine the expression of any regulator, which is very much related with this phage (S and G_2_/M) of cell cycle. We know that CyclinD1 can promote both G_0_/G_1_/S and S/G_2_/M progression [30]. Our western blot data shows that expression of CyclinD1 is increased in treated mice (Fig. 10A), for further confirmation we immunoprecipitated the cell lysate first with Cdk4 antibody, and the performed SDS-PAGE with anti CyclinD1 antibody, Fig. 10C indicated the complex formation of Cdk4-CyclinD1 which was an evidence of cell cycle progression. Data is representative of three independent experiments (p < 0.05).

## Discussion

In this study we tried to reveal the mechanistic details of Cadmium induced inflammation and proliferation in lung. An association between the development of cancer and inflammation has long been appreciated [31]. The chronic inflammatory states associated with infection and irritation may lead to environments that foster genomic lesions and tumor initiation [32]. The results of the present study showed that chronic exposure of cadmium compound induces lung cell proliferation which may be independent of lung inflammation. We hypothesized that cadmium exposure induces the inflammatory cytokines along with the cell proliferating factors in the lungs of mice.

We found that cadmium causes cell death at high concentration but at low level it is capable of inducing proliferation. Evidences are there indicate that low dose of cadmium can induce neoplastic transformation of human prostate epithelial cells [33]. Lung epithelial cells of cadmium treated (5 mg/Kg body weight) mice exhibits elevated level of cellular proliferation along with the accumulation of inflammatory molecules and cytokines. Therefore, in search of the mechanism behind it we found that cadmium induced the cellular signals to shift towards the proliferation as a whole, but prior to the development of cell proliferation cadmium initiated sever lung inflammation. There is a supportive evidence that, lung cell is able to become gradually resistant in response to cadmium [34]. We observed that IL-6 and inducible inflammatory enzyme Cox-2 elevated significantly in cadmium exposed mice. We also observed the expression level of TNFα, IL-1β, Hsp70, in cadmium treated lung cell (data not shown), but we did not find any significant change in their expression. That is why we mainly focused to see the role of cadmium on influencing the cellular expression of Cox-2, IL-6 and their down stream mediators, because of the fact that along with inflammation, these are the two known parameters of tumor development. Currently, Cox-2 inhibitors are being assessed in clinical trials for chemoprevention and as an adjuvant for conventional therapy in lung cancer [35]. Additionally, anti-IL-6 therapy has shown promising results in metastatic condition [36]. In the current study, we showed that elevated IL-6 and Cox-2 expression could be reduced by non steroidal anti-inflammatory drug like Ibuprofen. But at the same time molecular mechanisms of their regulation remains under observation (Data not shown here). Morphological differences between lungs of control and treated mice clearly suggest the development of edema. In inflammatory setting the inducible Cox-2 are detected in various reports [37]. We showed it also through ELISA and immunohistochemistry. In our consideration lung cells switch on such signaling which helps to proliferate against the stressed environment. It is already suggested by various scientists that IL-6-activated Janus kinase which leads to the activation of signal transducer and activator of transcription (STAT) and Akt signaling cascades [38]. There are supportive evidences that cadmium increases IL-6 production [39] and Akt activation in cancer cell [40]. On the other hand IL-6 induced ICAM-1 expression is mediated via JAK/STAT signaling pathway in which STAT3 phosphorylation followed by its binding to IRE which are in the promoter of cell cycle-related genes including CyclinD1[41]. Constitutive activation of STAT3 signaling contributes to oncogenesis by preventing apoptosis and enhancing cell proliferation. Moreover, Cox-2 dependent expression of IL-6 has been implicated in STAT3 activation and IL-6-dependent STAT3 activation has been shown to increase angiogenesis in several cancers [42]. We showed that though Ibuprofen reduces the expression level of Cox-2 and IL-6, it could not prevent the expression of p-STAT3 and p-Akt and CyclinD1. So, due to chronic exposure, cadmium promoted inflammation independent of signaling towards. Our flowcytometric results suggest cell cycle progression. It has been reported that STAT3 activation up regulates target genes, such as CyclinD1, that leads to cell cycle progression or prevention of apoptosis and STAT3 inhibition results in the down-regulation of CyclinD1[43]. As we know that CyclinD1 is responsible for G_0_/G_1_/S and S/G_2_/M transition [30], therefore, we have correlated both of the background information to test the expression of CyclinD1 and found that CyclinD1 was upregulated in lung cell after cadmium treatment, suggesting these pathways may operate in our system. Collectively, these findings suggest an important role for IL-6, Cox-2, STAT3, Akt and CyclinD1 in cadmium induced inflammation and lung cell proliferation.

We exposed mice to different concentrations of Cadmium and found that low levels of Cadmium (5 mg) consistently increased cell viability but higher levels of cadmium inevitably led to cell death with same exposure. A similar biphasic response has been reported previously by in vitro study [25]. So it is the first in vivo study which might provide a mechanistic explanation for Cadmium-induced cell proliferation independent of inflammation in normal lung cells. Many of the observations described cells are adapted when cadmium exposure continued for a longer period [44]. Similar observations of chronic pulmonary inflammation reported in cigarette smoke exposed mice [45]. We proved that for the first time that cadmium exposure promotes pulmonary inflammation but this may not be the cause of lung cell proliferation.

## Conclusion

These data provide a new insight into the relation between chronic inflammation and cell proliferation in vivo. Involvement of different inflammatory, signaling and cell cycle regulatory molecules in cadmium induced mice lung cell require further investigation. Our experimental model have both limitations and advantages. However, one major point should be taken into consideration before comparing our results obtained in mice model with human is that, though minute amount of cadmium deposit in lung if administered intraperitoneally or through contaminating food, it can still induces inflammation and proliferation due to persistent presence in lung cell but this two events may occur independently. Further in vitro studies in progress to delineate the cross-talk of inflammation and proliferation by cadmium.

## Abbreviations

Cd: cadmium; Cox-2: Cyclooxygenase-2; STAT3: signal transducer and activator of transcription factor 3; IL-6: Interleukin-6; i.p: intraperitonial; ELISA: enzyme-linked immunosorbent assay; SEM: scanning electron microscopy; MMP: matrix metalloproteinases; NSAID: non steroidal anti-inflammatory drug.

## Competing interests

The authors declare that they have no competing interests.

## Authors' contributions

SK is the head player of designing all these experiments. SS helped in histology, ELISA and immunohistochemistry. SC contributed by his analysis. SM and TK helped in animal handling western blot. AB designed and supervised all the experiments. All authors read and approved the final manuscript.
